# Features of Japanese juvenile spondyloarthritis patients in our hospital

**DOI:** 10.1186/1546-0096-12-S1-P222

**Published:** 2014-09-17

**Authors:** Nami Okamoto, Keisuke Shindo, Kosuke Shabana, Takuji Murata, Hiroshi Tamai

**Affiliations:** 1 pediatric, Osaka Medical College, Takatsuki-city, Japan

## Introduction

Spondyloarthritis (SpA) is thought to be very rare in Japan, because possession rate of HLA-B27 in healthy people is 0.4% and it is significantly lower than that of other countries (5%). This makes it difficult for Japanese physicians to diagnose SpA early. Since 10-20% of SpA patients experience symptoms in their childhood, it is very important to distinguish them from other chronic arthritis patients for pediatric rheumatologists.

## Objectives

We investigated the actual situation among Japanese patients with chronic arthritis and the features of juvenile SpA patients in our hospital.

## Methods

We checked age at disease onset, age at diagnosis, sex, hight SD, HLA and treatment.

## Results

There are eleven SpA cases (14.4% of children with chronic arthritis in our hospital), containing 5 axial SpA and 6 peripheral SpA cases. One psoriatic arthritis patient and one SAPHO patient are included in peripheral SpA. Male to female is 6 to 5. Age at disease onset ranges from 1.5 to 13.6 years old (median: 8.0 years old). The duration from disease onset to diagnosed age ranges from 0.6 to 11 years (median: 1.8 years). HLA-B27 possession rate is 30% and much higher than that of Japanese population. Possession rates of HLA-A2 and A24 are 50% respectively and also higher than those (25% respectively). Two peripheral SpA patients have family history. All three axial SpA patients with longer duration between onset and diagnosis than 1 year accompanied short statue (from -2.4 to -5.5 SD). All patients are treated with NSAIDs and nine patients are prescribed methotrexate (MTX). All five axial SpA patients need TNF inhibitors (etanercept for 2 cases and adalimumab for 3 cases) and three peripheral SpA patients need TNF inhibitor (adalimumab) to control disease activity. Sulfasalazine is adopted in two peripheral cases. Prevalence of SpA was originally reported to be 0.0095% and be significantly low in Japan. However Fujita recently reported that it amounted to 0.2% in their population survey held at Wakayama prefecture and insisted that real prevalence of Japanese SpA would be almost same as other countries. Low degree of recognition for SpA among Japanese physicians and difference of genetic background might account for this discrepancy. Similarly, juvenile SpA patients are thought to be very rare in Japan. Takei reported that enthesis-related and psoriatic types among JIA amount to be 1.6%, based on data from national medical profit project for chronic pediatric diseases. Our data shows that juvenile SpA patients amount to 14.4% of children with chronic arthritis in our hospital and does not differ big from other countries. One reason for this result could be that our hospital is one of specialized centers for pediatric rheumatism with trained medical specialists and diagnostic accuracy is higher. HLA-B27 negative patients or peripheral SpA patients are generally diagnosed inadequately. The other reason is that patients without apparent joint involvement tend to be referred to our facilities, so there can be some statistic bias. However, it is interesting that there are some numbers of SpA patients in Japan, the country at where prevalence of HLA-B27 does not high.

## Conclusion

We hope that survey on juvenile SpA advances since now and pathology of HLA-B27 negative patients will be disclosed all over the world.

## Disclosure of interest

None declared.

